# Supplementary material for: Laser ablation of Dbx1 neurons in the pre-Bötzinger complex stops inspiratory rhythm and impairs output in neonatal mice
Source: eLife. 2014 Jul 15;3:e03427. doi: 10.7554/eLife.03427 (PMC4129438; doi:10.7554/eLife.03427)
Supplement: Supplementary file 1. — Numerical simulations of Dbx1 neurons in model preBötC networks subjected to cumulative laser ablation experiments. Erdős-Rényi random directed graphs G(n,p) were populated with Rubin-Hayes preBötC neuron models at each node, and their links were described by excitatory synapses, as described for Figure 6 and Figure 6—figure supplement 1 above. In numerical simulations, the resulting network models with very high probability of generating rhythm and respiratory-like cycle period (∼4 s, indicated by asterisks in Figure 6A and Figure 6—figure supplement 1) were subjected to piecewise cumulative ablation protocols like slice experiments (Figures 2–4). The parameters describing the model networks (number of neurons n and synaptic connection probability p) are listed below in columns 1 and 2. Each ablation experiment was simulated five or more times. The maximum period (in s) and cumulative ablation tally (unitless) required to stop the rhythm are listed in the table for each individual realization of the network model along with average values for these characteristic measures. The networks deemed to be most representative of the preBötC, i.e., (n,p) = (320, 0.1375), (330, 0.125), (340, 0.125) were simulated 16 times each. DOI: http://dx.doi.org/10.7554/eLife.03427.018 [file elife03427s001.docx]

# SUPPLEMENTARY FILE 1

Numerical simulations of Dbx1 neurons in model preBötC networks subjected to cumulative laser ablation experiments. Erdős-Rényi random directed graphs G(*n,p*) were populated with Rubin-Hayes preBötC neuron models at each node, and their links were described by excitatory synapses, as described for Figure S5 above. In numerical simulations, the resulting network models with very high probability of generating rhythm and respiratory-like cycle period (~4 s, indicated by asterisks in Figures 6A and Figure 6 – figure supplement 1) were subjected to piecewise cumulative ablation protocols like slice experiments (Figures 2-4). The parameters describing the model networks (number of neurons *n* and synaptic connection probability *p*) are listed below in columns 1 and 2. Each ablation experiment was simulated five or more times. The maximum period (in sec) and cumulative ablation tally (unitless) required to stop the rhythm are listed in the table for each individual realization of the network model along with average values for these characteristic measures. The networks deemed to be most representative of the preBötC, i.e., (*n,p*) = (320, 0.1375), (330, 0.125), (340, 0.125) were simulated 16 times each.

| *n* | *p* | Sim. num. | Max. period (s) | Ablation tally |
| --- | --- | --- | --- | --- |
| 220 | 0.2 | 1 | 15.35 | 51 |
|  |  | 2 | 55.09 | 44 |
|  |  | 3 | 15.03 | 14 |
|  |  | 4 | 7.96 | 19 |
|  |  | 5 | 13.61 | 42 |
|  |  | 6 | 71.27 | 16 |
|  |  | average | 29.72 | 31 |
| 230 | 0.1875 | 1 | 13.12 | 11 |
|  |  | 2 | 20.18 | 42 |
|  |  | 3 | 16.52 | 36 |
|  |  | 4 | 30.97 | 50 |
|  |  | 5 | 20.74 | 30 |
|  |  | average | 20.31 | 34 |
| 240 | 0.175 | 1 | 18.60 | 21 |
|  |  | 2 | 19.09 | 29 |
|  |  | 3 | 25.10 | 26 |
|  |  | 4 | 17.48 | 37 |
|  |  | 5 | 24.18 | 28 |
|  |  | 6 | 14.71 | 46 |
|  |  | average | 19.86 | 32 |
| 250 | 0.1625 | 1 | 46.81 | 34 |
|  |  | 2 | 15.55 | 13 |
|  |  | 3 | 19.21 | 11 |
|  |  | 4 | 39.07 | 9 |
|  |  | 5 | 9.75 | 22 |
|  |  | 6 | 16.28 | 24 |
|  |  | average | 24.45 | 19 |
| 260 | 0.1625 | 1 | 63.51 | 35 |
|  |  | 2 | 13.76 | 27 |
|  |  | 3 | 25.45 | 27 |
|  |  | 4 | 27.88 | 14 |
|  |  | 5 | 28.47 | 38 |
|  |  | 6 | 111.55 | 48 |
|  |  | average | 45.10 | 32 |
| 270 | 0.1625 | 1 | 15.69 | 27 |
|  |  | 2 | 20.61 | 39 |
|  |  | 3 | 45.17 | 16 |
|  |  | 4 | 13.15 | 55 |
|  |  | 5 | 8.87 | 42 |
|  |  | average | 20.70 | 36 |
| 280 | 0.15 | 1 | 21.77 | 14 |
|  |  | 2 | 8.76 | 21 |
|  |  | 3 | 28.49 | 32 |
|  |  | 4 | 13.04 | 61 |
|  |  | 5 | 17.17 | 49 |
|  |  | average | 17.85 | 35 |
| 290 | 0.1375 | 1 | 17.62 | 23 |
|  |  | 2 | 15.68 | 26 |
|  |  | 3 | 87.94 | 37 |
|  |  | 4 | 34.11 | 37 |
|  |  | 5 | 20.21 | 15 |
|  |  | average | 35.11 | 27 |
| 300 | 0.1375 | 1 | 21.53 | 43 |
|  |  | 2 | 19.46 | 23 |
|  |  | 3 | 14.61 | 26 |
|  |  | 4 | 15.19 | 34 |
|  |  | 5 | 70.49 | 38 |
|  |  | 6 | 50.33 | 9 |
|  |  | average | 31.94 | 29 |
| 310 | 0.1375 | 1 | 18.39 | 59 |
|  |  | 2 | 23.89 | 49 |
|  |  | 3 | 43.97 | 30 |
|  |  | 4 | 38.14 | 48 |
|  |  | 5 | 9.45 | 19 |
|  |  | 6 | 9.33 | 50 |
|  |  | average | 23.86 | 43 |
| 320 | 0.1375 | 1 | 12.81 | 49 |
|  |  | 2 | 21.32 | 42 |
|  |  | 3 | 7.94 | 31 |
|  |  | 4 | 17.72 | 45 |
|  |  | 5 | 11.97 | 65 |
|  |  | 6 | 25.15 | 63 |
|  |  | 7 | 31.57 | 71 |
|  |  | 8 | 50.15 | 53 |
|  |  | 9 | 22.12 | 48 |
|  |  | 10 | 23.23 | 79 |
|  |  | 11 | 49.95 | 92 |
|  |  | 12 | 19.80 | 53 |
|  |  | 13 | 18.83 | 69 |
|  |  | 14 | 29.54 | 52 |
|  |  | 15 | 28.52 | 65 |
|  |  | 16 | 18.17 | 52 |
|  |  | average | 24.30 | 58 |
| 330 | 0.125 | 1 | 15.29 | 48 |
|  |  | 2 | 12.39 | 28 |
|  |  | 3 | 24.97 | 54 |
|  |  | 4 | 73.85 | 36 |
|  |  | 5 | 85.54 | 19 |
|  |  | 6 | 43.81 | 25 |
|  |  | 7 | 15.05 | 25 |
|  |  | 8 | 17.97 | 43 |
|  |  | 9 | 22.10 | 28 |
|  |  | 10 | 35.63 | 32 |
|  |  | 11 | 21.56 | 43 |
|  |  | 12 | 48.62 | 51 |
|  |  | 13 | 14.34 | 35 |
|  |  | 14 | 33.15 | 64 |
|  |  | 15 | 14.56 | 52 |
|  |  | 16 | 14.64 | 51 |
|  |  | average | 30.84 | 40 |
| 340 | 0.125 | 1 | 12.45 | 57 |
|  |  | 2 | 35.49 | 79 |
|  |  | 3 | 14.94 | 61 |
|  |  | 4 | 22.28 | 83 |
|  |  | 5 | 28.62 | 50 |
|  |  | 6 | 57.36 | 61 |
|  |  | 7 | 17.91 | 78 |
|  |  | 8 | 13.34 | 59 |
|  |  | 9 | 20.10 | 63 |
|  |  | 10 | 39.85 | 30 |
|  |  | 11 | 13.60 | 39 |
|  |  | 12 | 14.41 | 26 |
|  |  | 13 | 24.76 | 61 |
|  |  | 14 | 18.01 | 17 |
|  |  | 15 | 19.97 | 39 |
|  |  | 16 | 90.11 | 25 |
|  |  | average | 27.70 | 52 |
| 350 | 0.125 | 1 | 9.62 | 91 |
|  |  | 2 | 10.96 | 85 |
|  |  | 3 | 51.36 | 34 |
|  |  | 4 | 26.42 | 50 |
|  |  | 5 | 14.53 | 81 |
|  |  | 6 | 9.17 | 50 |
|  |  | average | 20.35 | 65 |
| 360 | 0.1125 | 1 | 24.99 | 28 |
|  |  | 2 | 58.05 | 31 |
|  |  | 3 | 60.49 | 46 |
|  |  | 4 | 23.83 | 46 |
|  |  | 5 | 42.22 | 24 |
|  |  | 6 | 20.19 | 18 |
|  |  | average | 38.30 | 32 |
| 370 | 0.1125 | 1 | 14.20 | 43 |
|  |  | 2 | 10.18 | 52 |
|  |  | 3 | 41.49 | 56 |
|  |  | 4 | 36.23 | 77 |
|  |  | 5 | 40.18 | 34 |
|  |  | 6 | 21.65 | 39 |
|  |  | average | 27.32 | 50 |
| 380 | 0.1125 | 1 | 13.18 | 67 |
|  |  | 2 | 96.25 | 22 |
|  |  | 3 | 36.81 | 59 |
|  |  | 4 | 8.41 | 49 |
|  |  | 5 | 18.27 | 39 |
|  |  | 6 | 20.58 | 49 |
|  |  | average | 32.25 | 48 |
| 390 | 0.1125 | 1 | 69.82 | 56 |
|  |  | 2 | 26.87 | 49 |
|  |  | 3 | 33.97 | 67 |
|  |  | 4 | 16.32 | 77 |
|  |  | 5 | 14.41 | 84 |
|  |  | average | 32.28 | 67 |
