# Supplementary material for: Laser ablation of Dbx1 neurons in the pre-Bötzinger complex stops inspiratory rhythm and impairs output in neonatal mice
Source: eLife. 2014 Jul 15;3:e03427. doi: 10.7554/eLife.03427 (PMC4129438; doi:10.7554/eLife.03427)
Supplement: Supplementary file 2. — Discrete network simulations. As in the main text and the table above, parameters (n,p) represent the number of constituent neurons and connection probability. Here the networks are Erdős-Rényi static directed random graphs G(n,p) (as in Figure 6 and Figure 6—supplement 1); that is, the nodes are not populated with dynamical models and the interconnections between nodes are simply static directed links (rather than dynamical synapses). Each network (static graph) below was subjected to 100 random deletions. During the ablation sequence, we computed the following global network metrics: K-core, the number of strongly connected components (SCC), and local network metrics: local cluster coefficient, closeness centrality, and betweenness centrality. The initial (first) and final (last) values for the global and local measures are plotted side by side in the appropriate columns below. We also computed the initial in- and out-degree (i.e., the average number of directed connections in and out), percentage drop in the final average in-degree, and the percentage-drop of the final average out-degree. The ‘Ave.’ row reports average change (in percent) for K-core, SCC, cluster coefficient, closeness centrality, and betweenness centrality, as well as the average in- and out-degree for initial and final states of the network. Definitions for the characteristic measures are elaborated in the ‘Materials and methods’. DOI: http://dx.doi.org/10.7554/eLife.03427.019 [file elife03427s002.docx]

# SUPPLEMENTARY FILE 2

Discrete network simulations. As in the main text and the table above, parameters (*n,p*) represent the number of constituent neurons and connection probability. Here the networks are Erdős-Rényi static directed random graphs G(*n,p*) (as in Figures 6 and Figure 6 – supplement 1); that is, the nodes are not populated with dynamical models and the interconnections between nodes are simply static directed links (rather than dynamical synapses). Each network (static graph) below was subjected to 100 random deletions. During the ablation sequence we computed the following global network metrics: K-core, the number of strongly connected components (SCC), and local network metrics: local cluster coefficient, closeness centrality, and betweenness centrality. The initial (first) and final (last) values for the global and local measures are plotted side by side in the appropriate columns below. We also computed the initial in- and out-degree (i.e., the average number of directed connections in and out), percentage drop in the final average in-degree, and the percentage-drop of the final average out-degree. The “Ave.” row reports average change (in percent) for K-core, SCC, cluster coefficient, closeness centrality, and betweenness centrality, as well as the average in- and out-degree for initial and final states of the network. Definitions for the characteristic measures are elaborated in the Materials and methods.

| *n* | *p* | run | K-core  (first, last) | SCC | Local cluster coefficient | Closeness centrality | Betweenness  centrality | Initial degree  (in & out) | Final in-degree (Percentage drop) | Final out-degree (Percentage drop) |
| --- | --- | --- | --- | --- | --- | --- | --- | --- | --- | --- |
| 220 | 0.2 | 1 | 29 20 | 1 1 | 0.16 0.23 | 0.55 0.56 | 0.0028 0.0048 | 44.11 | 34.52 | 33.78 |
|  |  | 2 | 27 21 | 1 1 | 0.25 0.16 | 0.55 0.56 | 0.0030 0.0058 | 44.15 | 35.68 | 33.82 |
|  |  | 3 | 30 28 | 1 1 | 0.15 0.28 | 0.54 0.57 | 0.0026 0.0037 | 44.02 | 41.52 | 41.26 |
|  |  | 4 | 29 25 | 1 1 | 0.17 0.19 | 0.55 0.56 | 0.0032 0.0042 | 43.60 | 40.23 | 39.40 |
|  |  | 5 | 30 21 | 1 1 | 0.28 0.24 | 0.56 0.57 | 0.0032 0.0044 | 43.84 | 35.80 | 35.26 |
|  |  | 6 | 29 26 | 1 1 | 0.26 0.24 | 0.57 0.56 | 0.0042 0.0038 | 43.81 | 41.12 | 40.44 |
|  |  | Ave. | 5.5 (19.0%) | 0 | 0.06 (27.5%) | 0.01 (1.7%) | 0.0014 (44.2%) | 43.92 | 38.15 (13.2%) | 37.33 (15.0%) |
| 230 | 0.1875 | 1 | 25 25 | 1 1 | 0.22 0.17 | 0.53 0.52 | 0.0037 0.0035 | 43.00 | 41.37 | 41.09 |
|  |  | 2 | 28 22 | 1 1 | 0.19 0.17 | 0.56 0.55 | 0.0034 0.0046 | 43.27 | 35.91 | 34.89 |
|  |  | 3 | 29 23 | 1 1 | 0.19 0.18 | 0.56 0.56 | 0.0044 0.0047 | 43.08 | 36.84 | 36.26 |
|  |  | 4 | 28 21 | 1 1 | 0.25 0.17 | 0.57 0.56 | 0.0050 0.0050 | 43.10 | 34.08 | 32.94 |
|  |  | 5 | 28 22 | 1 1 | 0.15 0.26 | 0.55 0.55 | 0.0034 0.0028 | 42.76 | 37.71 | 36.84 |
|  |  | Ave. | 5 (18.1%) | 0 | 0.06 (27.5%) | 0.01 (1.2%) | 0.0005 (12.6%) | 43.04 | 37.18 (13.6%) | 36.40 (15.4%) |
| 240 | 0.175 | 1 | 27 24 | 1 1 | 0.16 0.17 | 0.50 0.50 | 0.0039 0.0049 | 42.03 | 38.87 | 38.50 |
|  |  | 2 | 30 26 | 1 1 | 0.20 0.22 | 0.55 0.57 | 0.0033 0.0049 | 42.10 | 37.25 | 36.38 |
|  |  | 3 | 26 22 | 1 1 | 0.18 0.17 | 0.54 0.55 | 0.0026 0.0041 | 41.57 | 37.54 | 37.10 |
|  |  | 4 | 27 23 | 1 1 | 0.15 0.18 | 0.54 0.55 | 0.0025 0.0040 | 41.83 | 35.74 | 33.91 |
|  |  | 5 | 24 20 | 1 1 | 0.23 0.14 | 0.55 0.54 | 0.0025 0.0032 | 41.70 | 37.32 | 36.65 |
|  |  | 6 | 27 21 | 1 1 | 0.21 0.22 | 0.56 0.554 | 0.0037 0.0042 | 41.99 | 33.86 | 33.38 |
|  |  | Ave. | 4.2 (15.1%) | 0 | 0.03 (14.3%) | 0.01 (1.6%) | 0.0011 (35.7%) | 41.87 | 36.76 (12.2%) | 35.99 (14.0%) |
| 250 | 0.1625 | 1 | 25 22 | 1 1 | 0.17 0.21 | 0.48 0.47 | 0.0046 0.0041 | 40.92 | 35.74 | 34.96 |
|  |  | 2 | 21 20 | 1 1 | 0.15 0.25 | 0.55 0.56 | 0.0039 0.0034 | 40.78 | 39.00 | 38.58 |
|  |  | 3 | 21 20 | 1 1 | 0.22 0.18 | 0.55 0.55 | 0.0034 0.0041 | 39.67 | 38.07 | 37.58 |
|  |  | 4 | 24 24 | 1 1 | 0.15 0.10 | 0.54 0.54 | 0.0025 0.0038 | 40.30 | 39.21 | 38.98 |
|  |  | 5 | 20 20 | 1 1 | 0.22 0.18 | 0.56 0.55 | 0.0046 0.0042 | 40.87 | 37.70 | 36.47 |
|  |  | 6 | 27 25 | 1 1 | 0.18 0.19 | 0.55 0.55 | 0.0034 0.0034 | 40.49 | 36.78 | 36.42 |
|  |  | Ave. | 1.1 (4.8%) | 0 | 0.05 (25.6%) | 0.01 (1.2%) | 0.0006 (16.1%) | 40.50 | 37.75 (6.8%) | 37.16 (8.2%) |
| 260 | 0.1625 | 1 | 28 23 | 1 1 | 0.20 0.14 | 0.55 0.54 | 0.0036 0.0033 | 42.15 | 37.30 | 36.55 |
|  |  | 2 | 29 25 | 1 1 | 0.15 0.12 | 0.54 0.54 | 0.0035 0.0034 | 42.28 | 38.18 | 37.69 |
|  |  | 3 | 24 24 | 1 1 | 0.17 0.14 | 0.53 0.54 | 0.0017 0.0034 | 42.44 | 38.22 | 36.92 |
|  |  | 4 | 30 26 | 1 1 | 0.21 0.20 | 0.54 0.55 | 0.0024 0.0033 | 42.20 | 40.42 | 39.61 |
|  |  | 5 | 26 21 | 1 1 | 0.19 0.14 | 0.55 0.55 | 0.0037 0.0045 | 42.25 | 36.73 | 36.28 |
|  |  | 6 | 29 24 | 1 1 | 0.21 0.18 | 0.56 0.54 | 0.0046 0.0026 | 42.81 | 35.29 | 33.72 |
|  |  | Ave. | 3.8 (13.7%) | 0 | 0.03 (17.9%) | 0.01 (2.0%) | 0.001 (30.8%) | 42.35 | 37.69 (11.0%) | 36.79 (13.1%) |
| 270 | 0.1625 | 1 | 26 22 | 1 1 | 0.09 0.13 | 0.53 0.54 | 0.0021 0.0028 | 44.26 | 40.16 | 38.72 |
|  |  | 2 | 26 25 | 1 1 | 0.16 0.19 | 0.55 0.54 | 0.0032 0.0022 | 43.87 | 37.77 | 37.47 |
|  |  | 3 | 27 24 | 1 1 | 0.21 0.14 | 0.55 0.55 | 0.0025 0.0041 | 43.97 | 41.91 | 41.22 |
|  |  | 4 | 28 21 | 1 1 | 0.18 0.26 | 0.55 0.55 | 0.0032 0.0030 | 44.07 | 35.08 | 34.44 |
|  |  | 5 | 25 21 | 1 1 | 0.22 0.17 | 0.55 0.54 | 0.0027 0.0024 | 44.06 | 37.61 | 44.06 |
|  |  | Ave. | 3.8 (14.4%) | 0 | 0.05 (31.3%) | 0.01 (1.6%) | 0.0008 (29.2%) | 44.05 | 38.51 (12.6%) | 39.18 (11.0%) |
| 280 | 0.15 | 1 | 25 25 | 1 1 | 0.12 0.17 | 0.50 0.50 | 0.0039 0.0031 | 41.88 | 40.15 | 39.76 |
|  |  | 2 | 25 25 | 1 1 | 0.14 0.16 | 0.54 0.55 | 0.0031 0.0036 | 41.98 | 38.98 | 38.58 |
|  |  | 3 | 25 21 | 1 1 | 0.16 0.13 | 0.54 0.54 | 0.0028 0.0037 | 41.74 | 37.30 | 36.39 |
|  |  | 4 | 24 20 | 1 1 | 0.18 0.14 | 0.54 0.54 | 0.0023 0.0036 | 42.06 | 33.14 | 32.06 |
|  |  | 5 | 25 20 | 1 1 | 0.16 0.18 | 0.55 0.55 | 0.0043 0.0043 | 42.00 | 35.13 | 33.64 |
|  |  | Ave. | 2.6 (10.5%) | 0 | 0.03 (20.2%) | 0.00 (0.4%) | 0.0007 (21.3%) | 41.93 | 36.94 (11.9%) | 36.09 (13.9%) |
| 290 | 0.1375 | 1 | 24 22 | 1 1 | 0.19 0.20 | 0.49 0.48 | 0.0043 0.0040 | 39.74 | 36.97 | 36.69 |
|  |  | 2 | 23 21 | 1 1 | 0.16 0.19 | 0.54 0.55 | 0.0026 0.0033 | 39.43 | 36.32 | 36.29 |
|  |  | 3 | 22 20 | 1 1 | 0.07 0.11 | 0.51 0.53 | 0.0019 0.0034 | 39.91 | 35.02 | 34.13 |
|  |  | 4 | 22 17 | 1 1 | 0.11 0.16 | 0.53 0.54 | 0.0025 0.0033 | 39.59 | 34.65 | 33.64 |
|  |  | 5 | 20 20 | 1 1 | 0.14 0.17 | 0.54 0.54 | 0.0032 0.0035 | 39.70 | 37.79 | 37.49 |
|  |  | Ave. | 2.2 (9.9%) | 0 | 0.03 (24.3%) | 0.01 (1.4%) | 0.0007 (24.1%) | 39.67 | 36.15 (8.9%) | 35.65 (10.1%) |
| 300 | 0.1375 | 1 | 26 21 | 1 1 | 0.14 0.12 | 0.46 0.45 | 0.0032 0.0047 | 41.56 | 35.81 | 34.34 |
|  |  | 2 | 22 21 | 1 1 | 0.14 0.17 | 0.55 0.54 | 0.0050 0.0033 | 41.23 | 38.10 | 37.69 |
|  |  | 3 | 23 22 | 1 1 | 0.12 0.17 | 0.54 0.55 | 0.0034 0.0041 | 41.54 | 37.92 | 36.74 |
|  |  | 4 | 23 22 | 1 1 | 0.09 0.12 | 0.54 0.54 | 0.0038 0.0039 | 41.20 | 36.70 | 34.62 |
|  |  | 5 | 27 22 | 1 1 | 0.16 0.10 | 0.54 0.53 | 0.0032 0.0033 | 41.00 | 36.16 | 35.14 |
|  |  | 6 | 26 25 | 1 1 | 0.10 0.14 | 0.53 0.54 | 0.0028 0.0032 | 41.31 | 40.16 | 40.02 |
|  |  | Ave. | 2.3 (9.4%) | 0 | 0.04 (31.4%) | 0.01 (1.7%) | 0.0008 (22.4%) | 41.31 | 37.48 (9.3%) | 36.43 (11.8%) |
| 310 | 0.1375 | 1 | 26 20 | 1 1 | 0.12 0.18 | 0.45 0.44 | 0.0049 0.0067 | 42.82 | 35.11 | 34.45 |
|  |  | 2 | 24 21 | 1 1 | 0.17 0.17 | 0.54 0.54 | 0.0022 0.0029 | 42.34 | 35.76 | 35.93 |
|  |  | 3 | 23 21 | 1 1 | 0.15 0.15 | 0.54 0.55 | 0.0036 0.0044 | 42.75 | 38.77 | 38.33 |
|  |  | 4 | 28 20 | 1 1 | 0.13 0.13 | 0.54 0.54 | 0.0028 0.0043 | 42.62 | 35.96 | 35.15 |
|  |  | 5 | 25 24 | 1 1 | 0.16 0.18 | 0.53 0.54 | 0.0022 0.0032 | 42.46 | 40.06 | 39.16 |
|  |  | 6 | 24 21 | 1 1 | 0.13 0.09 | 0.53 0.52 | 0.0023 0.0025 | 42.30 | 35.40 | 33.98 |
|  |  | Ave. | 3.8 (15.2%) | 0 | 0.02 (15.0%) | 0.01 (1.3%) | 0.001 (33.3%) | 42.55 | 36.84 (13.4%) | 36.17 (15.0%) |
| 320 | 0.1375 | 1 | 26 22 | 1 1 | 0.18 0.13 | 0.44 0.42 | 0.0034 0.0070 | 43.89 | 37.58 | 37.65 |
|  |  | 2 | 30 25 | 1 1 | 0.15 0.12 | 0.54 0.54 | 0.0027 0.0035 | 44.05 | 38.36 | 36.93 |
|  |  | 3 | 29 26 | 1 1 | 0.16 0.14 | 0.55 0.53 | 0.0035 0.0025 | 44.34 | 40.19 | 39.63 |
|  |  | 4 | 28 18 | 1 1 | 0.12 0.21 | 0.53 0.54 | 0.0024 0.0028 | 44.00 | 37.95 | 36.27 |
|  |  | 5 | 27 21 | 1 1 | 0.15 0.10 | 0.54 0.52 | 0.0024 0.0019 | 43.86 | 35.47 | 35.07 |
|  |  | 6 | 31 24 | 1 1 | 0.12 0.08 | 0.53 0.53 | 0.0022 0.0038 | 43.94 | 35.11 | 33.94 |
|  |  | 7 | 27 20 | 1 1 | 0.13 0.13 | 0.53 0.54 | 0.0023 0.0039 | 44.15 | 34.57 | 32.73 |
|  |  | 8 | 26 23 | 1 1 | 0.15 0.10 | 0.54 0.53 | 0.0024 0.0038 | 44.28 | 37.33 | 36.09 |
|  |  | 9 | 22 20 | 1 1 | 0.11 0.18 | 0.53 0.54 | 0.0019 0.0029 | 43.97 | 37.53 | 36.71 |
|  |  | 10 | 30 22 | 1 1 | 0.16 0.15 | 0.54 0.54 | 0.0032 0.0036 | 43.98 | 33.27 | 32.13 |
|  |  | 11 | 27 17 | 1 1 | 0.12 0.16 | 0.53 0.54 | 0.0023 0.0051 | 43.82 | 31.51 | 31.39 |
|  |  | 12 | 26 18 | 1 1 | 0.10 0.14 | 0.52 0.54 | 0.0020 0.0038 | 44.08 | 37.21 | 36.40 |
|  |  | 13 | 27 20 | 1 1 | 0.18 0.26 | 0.55 0.56 | 0.0031 0.0044 | 43.60 | 34.51 | 34.90 |
|  |  | 14 | 31 25 | 1 1 | 0.14 0.19 | 0.54 0.55 | 0.0030 0.0039 | 43.75 | 36.73 | 35.63 |
|  |  | 15 | 27 22 | 1 1 | 0.22 0.16 | 0.55 0.55 | 0.0026 0.0044 | 44.16 | 35.43 | 34.68 |
|  |  | 16 | 25 19 | 1 1 | 0.17 0.14 | 0.55 0.53 | 0.0029 0.0021 | 43.54 | 37.01 | 34.81 |
|  |  | Ave. | 6.1 | 0 | 0.00 (1.7%) | 0.00 (0.1%) | 0.0011 (40.4%) | 43.96 | 36.23 (17.6%) | 35.31 (19.7%) |
| 330 | 0.125 | 1 | 24 20 | 1 1 | 0.12 0.19 | 0.53 0.55 | 0.0022 0.0040 | 41.53 | 35.58 | 34.42 |
|  |  | 2 | 26 24 | 1 1 | 0.16 0.12 | 0.53 0.54 | 0.0021 0.0035 | 41.15 | 37.88 | 37.23 |
|  |  | 3 | 25 21 | 1 1 | 0.14 0.26 | 0.55 0.55 | 0.0042 0.0032 | 41.18 | 34.63 | 33.26 |
|  |  | 4 | 24 22 | 1 1 | 0.12 0.15 | 0.53 0.53 | 0.0021 0.0032 | 41.48 | 37.11 | 35.84 |
|  |  | 5 | 24 23 | 1 1 | 0.19 0.12 | 0.54 0.54 | 0.0025 0.0037 | 40.85 | 38.77 | 38.42 |
|  |  | 6 | 24 23 | 1 1 | 0.13 0.19 | 0.53 0.55 | 0.0025 0.0032 | 41.09 | 38.23 | 37.00 |
|  |  | 7 | 26 22 | 1 1 | 0.07 0.14 | 0.53 0.54 | 0.0028 0.0028 | 40.86 | 37.92 | 37.35 |
|  |  | 8 | 26 22 | 1 1 | 0.12 0.12 | 0.53 0.53 | 0.0022 0.0031 | 41.19 | 36.12 | 33.98 |
|  |  | 9 | 23 20 | 1 1 | 0.13 0.15 | 0.53 0.54 | 0.0026 0.0030 | 41.12 | 37.80 | 37.36 |
|  |  | 10 | 23 20 | 1 1 | 0.07 0.11 | 0.52 0.52 | 0.0021 0.0023 | 41.03 | 37.30 | 36.48 |
|  |  | 11 | 26 19 | 1 1 | 0.11 0.14 | 0.53 0.54 | 0.0029 0.0035 | 41.19 | 35.94 | 34.32 |
|  |  | 12 | 28 22 | 1 1 | 0.12 0.14 | 0.53 0.52 | 0.0020 0.0015 | 41.11 | 34.95 | 34.29 |
|  |  | 13 | 26 23 | 1 1 | 0.14 0.12 | 0.53 0.53 | 0.0024 0.0027 | 41.52 | 37.17 | 36.13 |
|  |  | 14 | 25 20 | 1 1 | 0.11 0.12 | 0.53 0.53 | 0.0029 0.0040 | 40.63 | 32.76 | 31.95 |
|  |  | 15 | 25 20 | 1 1 | 0.11 0.12 | 0.53 0.53 | 0.0032 0.0033 | 41.28 | 34.93 | 34.74 |
|  |  | 16 | 23 17 | 1 1 | 0.18 0.11 | 0.54 0.53 | 0.0022 0.0031 | 41.56 | 35.20 | 34.18 |
|  |  | Ave. | 3.8 | 0 | 0.02 (12.7%) | 0.00 (0.5%) | 0.0006 (22.5%) | 41.17 | 36.39 (11.6%) | 35.43 (13.9%) |
| 340 | 0.125 | 1 | 26 17 | 1 1 | 0.15 0.12 | 0.53 0.54 | 0.0022 0.0037 | 42.28 | 35.23 | 33.83 |
|  |  | 2 | 28 18 | 1 1 | 0.15 0.12 | 0.53 0.54 | 0.0016 0.0038 | 46.74 | 36.20 | 35.16 |
|  |  | 3 | 30 23 | 1 1 | 0.13 0.14 | 0.54 0.53 | 0.0023 0.0033 | 46.68 | 38.55 | 36.49 |
|  |  | 4 | 27 20 | 1 1 | 0.13 0.14 | 0.54 0.52 | 0.0022 0.0017 | 47.15 | 35.31 | 33.84 |
|  |  | 5 | 29 24 | 1 1 | 0.13 0.15 | 0.54 0.53 | 0.0030 0.0017 | 46.36 | 39.45 | 38.61 |
|  |  | 6 | 26 21 | 1 1 | 0.14 0.21 | 0.53 0.54 | 0.0021 0.0027 | 46.65 | 38.55 | 38.14 |
|  |  | 7 | 28 17 | 1 1 | 0.12 0.14 | 0.53 0.54 | 0.0019 0.0044 | 43.00 | 33.15 | 32.26 |
|  |  | 8 | 25 19 | 1 1 | 0.13 0.20 | 0.53 0.54 | 0.0022 0.0031 | 42.67 | 35.52 | 34.70 |
|  |  | 9 | 27 20 | 1 1 | 0.19 0.08 | 0.54 0.51 | 0.0021 0.0024 | 42.52 | 34.27 | 34.35 |
|  |  | 10 | 23 19 | 1 1 | 0.09 0.10 | 0.53 0.53 | 0.0029 0.0024 | 42.62 | 39.05 | 38.15 |
|  |  | 11 | 27 20 | 1 1 | 0.10 0.12 | 0.52 0.54 | 0.0021 0.0035 | 42.75 | 37.93 | 36.75 |
|  |  | 12 | 26 23 | 1 1 | 0.14 0.09 | 0.54 0.53 | 0.0036 0.0025 | 42.39 | 39.14 | 38.68 |
|  |  | 13 | 24 18 | 1 1 | 0.12 0.10 | 0.53 0.52 | 0.0025 0.0026 | 42.61 | 34.91 | 34.07 |
|  |  | 14 | 27 25 | 1 1 | 0.12 0.18 | 0.54 0.54 | 0.0029 0.0032 | 42.36 | 40.27 | 39.90 |
|  |  | 15 | 27 22 | 1 1 | 0.12 0.17 | 0.54 0.53 | 0.0029 0.0026 | 42.70 | 37.98 | 37.62 |
|  |  | 16 | 29 25 | 1 1 | 0.14 0.16 | 0.54 0.54 | 0.0025 0.0023 | 42.59 | 39.38 | 38.62 |
|  |  | Ave. | 6.1 | 0 | 0.01 (5.2%) | 0.00 (0.2%) | 0.0004 (17.7%) | 43.88 | 37.18 (15.3%) | 36.32 (17.2%) |
| 350 | 0.125 | 1 | 27 19 | 1 1 | 0.10 0.14 | 0.54 0.53 | 0.0034 0.0029 | 43.36 | 32.40 | 30.09 |
|  |  | 2 | 27 19 | 1 1 | 0.10 0.13 | 0.54 0.54 | 0.0036 0.0037 | 43.67 | 33.30 | 31.48 |
|  |  | 3 | 29 25 | 1 1 | 0.12 0.12 | 0.53 0.52 | 0.0021 0.0019 | 43.61 | 39.56 | 39.18 |
|  |  | 4 | 29 22 | 1 1 | 0.12 0.14 | 0.53 0.54 | 0.0026 0.0029 | 43.78 | 37.81 | 35.29 |
|  |  | 5 | 28 18 | 1 1 | 0.14 0.12 | 0.54 0.53 | 0.0030 0.0028 | 43.70 | 33.60 | 32.39 |
|  |  | 6 | 25 20 | 1 1 | 0.14 0.12 | 0.54 0.53 | 0.0028 0.0025 | 43.93 | 37.72 | 37.13 |
|  |  | Ave. | 7 (25.5%) | 0 | 0.02 (17.6%) | 0.01 (1.0%) | 0.0003 (10.3%) | 43.67 | 35.73 (18.2%) | 34.26 (21.6%) |
| 360 | 0.1125 | 1 | 24 22 | 1 1 | 0.08 0.12 | 0.52 0.53 | 0.0020 0.0023 | 40.66 | 37.85 | 37.18 |
|  |  | 2 | 26 24 | 1 1 | 0.20 0.10 | 0.53 0.53 | 0.0018 0.0035 | 40.65 | 37.23 | 36.61 |
|  |  | 3 | 22 19 | 1 1 | 0.09 0.12 | 0.53 0.54 | 0.0025 0.0042 | 40.71 | 35.61 | 35.88 |
|  |  | 4 | 24 20 | 1 1 | 0.13 0.12 | 0.54 0.53 | 0.0028 0.0026 | 40.42 | 35.30 | 34.12 |
|  |  | 5 | 24 22 | 1 1 | 0.14 014 | 0.54 0.54 | 0.0029 0.0034 | 40.42 | 37.81 | 37.47 |
|  |  | 6 | 22 21 | 1 1 | 0.13 0.12 | 0.53 0.53 | 0.0024 0.0028 | 40.02 | 38.31 | 37.78 |
|  |  | Ave. | 2.3 (9.7%) | 0 | 0.03 (22.1%) | 0.01 (1.0%) | 0.0008 (33.3%) | 40.48 | 37.02 (8.6%) | 36.51 (9.8%) |
| 370 | 0.1125 | 1 | 25 22 | 1 1 | 0.11 0.11 | 0.53 0.53 | 0.0023 0.0034 | 41.71 | 36.79 | 36.34 |
|  |  | 2 | 27 22 | 1 1 | 0.21 0.09 | 0.54 0.51 | 0.0018 0.0026 | 41.66 | 35.85 | 34.92 |
|  |  | 3 | 26 22 | 1 1 | 0.15 0.10 | 0.53 0.53 | 0.0024 0.0035 | 42.18 | 36.22 | 34.59 |
|  |  | 4 | 25 19 | 1 1 | 0.15 0.13 | 0.54 0.53 | 0.0031 0.0029 | 41.29 | 33.12 | 31.97 |
|  |  | 5 | 25 23 | 1 1 | 0.10 0.10 | 0.53 0.52 | 0.0029 0.0021 | 41.81 | 38.21 | 37.45 |
|  |  | 6 | 23 20 | 1 1 | 0.12 0.11 | 0.53 0.54 | 0.0020 0.0037 | 41.59 | 37.45 | 36.51 |
|  |  | Ave. | 3.8 (15.1%) | 0 | 0.03 (24.5%) | 0.01 (1.6%) | 0.0010 (41.4%) | 41.71 | 36.27 (13.0%) | 35.30 (15.4%) |
| 380 | 0.1125 | 1 | 27 22 | 1 1 | 0.13 0.12 | 0.53 0.53 | 0.0024 0.0030 | 43.24 | 35.50 | 33.78 |
|  |  | 2 | 24 22 | 1 1 | 0.12 0.21 | 0.53 0.53 | 0.0021 0.0020 | 42.32 | 39.94 | 39.12 |
|  |  | 3 | 27 22 | 1 1 | 0.16 0.08 | 0.53 0.52 | 0.0016 0.0024 | 42.65 | 36.10 | 35.21 |
|  |  | 4 | 29 20 | 1 1 | 0.10 0.13 | 0.53 0.54 | 0.0029 0.0034 | 42.35 | 37.35 | 36.87 |
|  |  | 5 | 28 23 | 1 1 | 0.09 0.12 | 0.52 0.53 | 0.0022 0.0024 | 43.01 | 38.99 | 38.15 |
|  |  | 6 | 25 21 | 1 1 | 0.08 0.10 | 0.53 0.53 | 0.0025 0.0031 | 42.61 | 37.51 | 36.69 |
|  |  | Ave. | 5 (18.8%) | 0 | 0.04 (34.8%) | 0.00 (0.8%) | 0.0006 (26.3%) | 42.70 | 37.56 (12.0%) | 36.64 (14.2%) |
| 390 | 0.1125 | 1 | 28 21 | 1 1 | 0.13 0.08 | 0.54 0.52 | 0.0022 0.0028 | 43.66 | 37.34 | 36.88 |
|  |  | 2 | 28 25 | 1 1 | 0.11 0.08 | 0.53 0.52 | 0.0024 0.0022 | 43.67 | 38.09 | 37.87 |
|  |  | 3 | 27 21 | 1 1 | 0.11 0.12 | 0.53 0.53 | 0.0024 0.0024 | 43.96 | 36.81 | 36.74 |
|  |  | 4 | 21 12 | 1 1 | 0.10 0.09 | 0.53 0.52 | 0.0029 0.0027 | 44.02 | 35.85 | 34.79 |
|  |  | 5 | 28 19 | 1 1 | 0.11 0.10 | 0.53 0.53 | 0.0026 0.0034 | 44.07 | 34.72 | 33.76 |
|  |  | Ave. | 6.8 (25.8%) | 0 | 0.02 (19.5%) | 0.01 (2.0%) | 0.0004 (16.0%) | 43.87 | 36.56 (16.7%) | 36.01 (17.9%) |
